# Activation of acetyl-CoA synthetase 2 mediates kidney injury in diabetic nephropathy

**DOI:** 10.1172/jci.insight.165817

**Published:** 2023-10-23

**Authors:** Jian Lu, Xue Qi Li, Pei Pei Chen, Jia Xiu Zhang, Liang Liu, Gui Hua Wang, Xiao Qi Liu, Ting Ting Jiang, Meng Ying Wang, Wen Tao Liu, Xiong Zhong Ruan, Kun Ling Ma

**Affiliations:** 1 Department of Nephrology, Nanjing Drum Tower Hospital, the Affiliated Hospital of Nanjing University Medical School, Nanjing, China.; 2 Institute of Nephrology, Zhongda Hospital, School of Medicine, Southeast University, Nanjing, China.; 3 Department of Intensive Care Unit, People’s Hospital Affiliated to Shandong First Medical University, Jinan, China.; 4 John Moorhead Research Laboratory, Department of Renal Medicine, University College London Medical School, Royal Free Campus, London, United Kingdom.; 5 Department of Nephrology, the Second Affiliated Hospital, School of Medicine, Zhejiang University, Hangzhou, China.

**Keywords:** Metabolism, Nephrology, Diabetes, Molecular biology

## Abstract

Albuminuria and podocyte injury are the key cellular events in the progression of diabetic nephropathy (DN). Acetyl-CoA synthetase 2 (ACSS2) is a nucleocytosolic enzyme responsible for the regulation of metabolic homeostasis in mammalian cells. This study aimed to investigate the possible roles of ACSS2 in kidney injury in DN. We constructed an ACSS2-deleted mouse model to investigate the role of ACSS2 in podocyte dysfunction and kidney injury in diabetic mouse models. In vitro, podocytes were chosen and transfected with ACSS2 siRNA and ACSS2 inhibitor and treated with high glucose. We found that ACSS2 expression was significantly elevated in the podocytes of patients with DN and diabetic mice. ACSS2 upregulation promoted phenotype transformation and inflammatory cytokine expression while inhibiting podocytes’ autophagy. Conversely, ACSS2 inhibition improved autophagy and alleviated podocyte injury. Furthermore, ACSS2 epigenetically activated raptor expression by histone H3K9 acetylation, promoting activation of the mammalian target of rapamycin complex 1 (mTORC1) pathway. Pharmacological inhibition or genetic depletion of ACSS2 in the streptozotocin-induced diabetic mouse model greatly ameliorated kidney injury and podocyte dysfunction. To conclude, ACSS2 activation promoted podocyte injury in DN by raptor/mTORC1-mediated autophagy inhibition.

## Graphical abstract

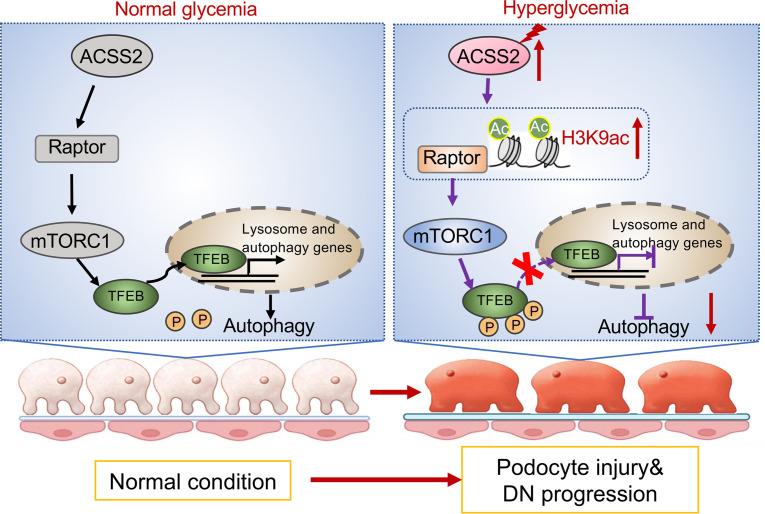

## Introduction

Diabetic nephropathy (DN) represents the most common secondary glomerular disorder causing end-stage renal disease (ESRD) (1). Hyperglycemia, a major pathological factor in diabetes mellitus, promotes DN development (2). However, blood glucose normalization is difficult, and renal protection effects of RAS inhibitors cannot completely prevent DN progression (3). The exact mechanisms by which hyperglycemia affects renal pathological processes are poorly characterized. Therefore, the development of new therapeutic regimens needs to identify new molecular mechanisms of DN. Podocytes are differentiated epithelial cells characterized by interdigitating foot processes covering the outer surface of glomerular capillaries (4).

Podocyte injury is a pivotal manifestation of diabetes mellitus. During the process, the podocyte undergoes different degrees of structural and functional damage ranging from hypertrophy, dedifferentiation, autophagy, apoptosis, and eventually detachment (5). The podocyte injury ultimately leads to massive albuminuria and the disease progression of proteinuria kidney disease. Unfortunately, irreversible and progressive podocyte injury is considered a surrogate for poor prognosis and ESRD. Therefore, new therapeutic methods and the discovery of novel molecular targets aimed at preventing podocyte injury have significant clinical therapeutic prospects.

Acetyl-CoA synthetase 2 (ACSS2), localized in the cytoplasm and nucleus of mammalian cells (6), was originally identified as an essential enzyme to convert acetate to acetyl-CoA (7), which is a central metabolic intermediate for transcriptional regulation and substrate for de novo lipogenesis (DNL) (8). Hepatocyte-specific depletion of ACSS2 decreased hepatic acetyl-CoA levels and DNL in obese mice, emphasizing the contribution of ACSS2 to the generation of fatty acid (9). In tumor cells, acetate activated pro-lipogenic genes through ACSS2 by increasing histone acetylation levels at these gene promoter regions (10). Accumulated evidence shows that ACSS2 is involved in multiple physiological and pathological processes, such as spatial memory (11), thermogenesis (12), lipogenesis (13), and oncogenesis (14). ACSS2 upregulation also functions as a vital regulator in cytomegalovirus infection (15) and glucose/lipid metabolic abnormality (16).

The main functional identity of ACSS2 is characterized by the modulation of histone acetylation. ACSS2-dependent acetylation of histone H3K9 or H3K27 is essential for the transcriptional expression of genes associated with autophagy and lysosomal biogenesis (13). Moreover, ACSS2 tightly correlates with poor prognosis in multiple cancers (17). ACSS2 facilitates the acetyl-CoA synthesis to support tumor survival and proliferative phenotype under metabolic stress conditions (6, 18, 19). However, the role of ACSS2 in the kidney and the underlying mechanisms remain largely unclear.

Therefore, this study aimed to evaluate the possible roles of ACSS2 in kidney injury in DN by using kidney tissues from biopsied patients with DN, a podocyte cell culture model, and diabetic mice with an intragastric injection of a specific ACSS2 inhibitor, streptozotocin-treated ACSS2-knockout (ACSS2-KO) mice.

## Results

### ACSS2 expression increases in the kidneys of patients with DN and diabetic mice.

**Figure 1:**
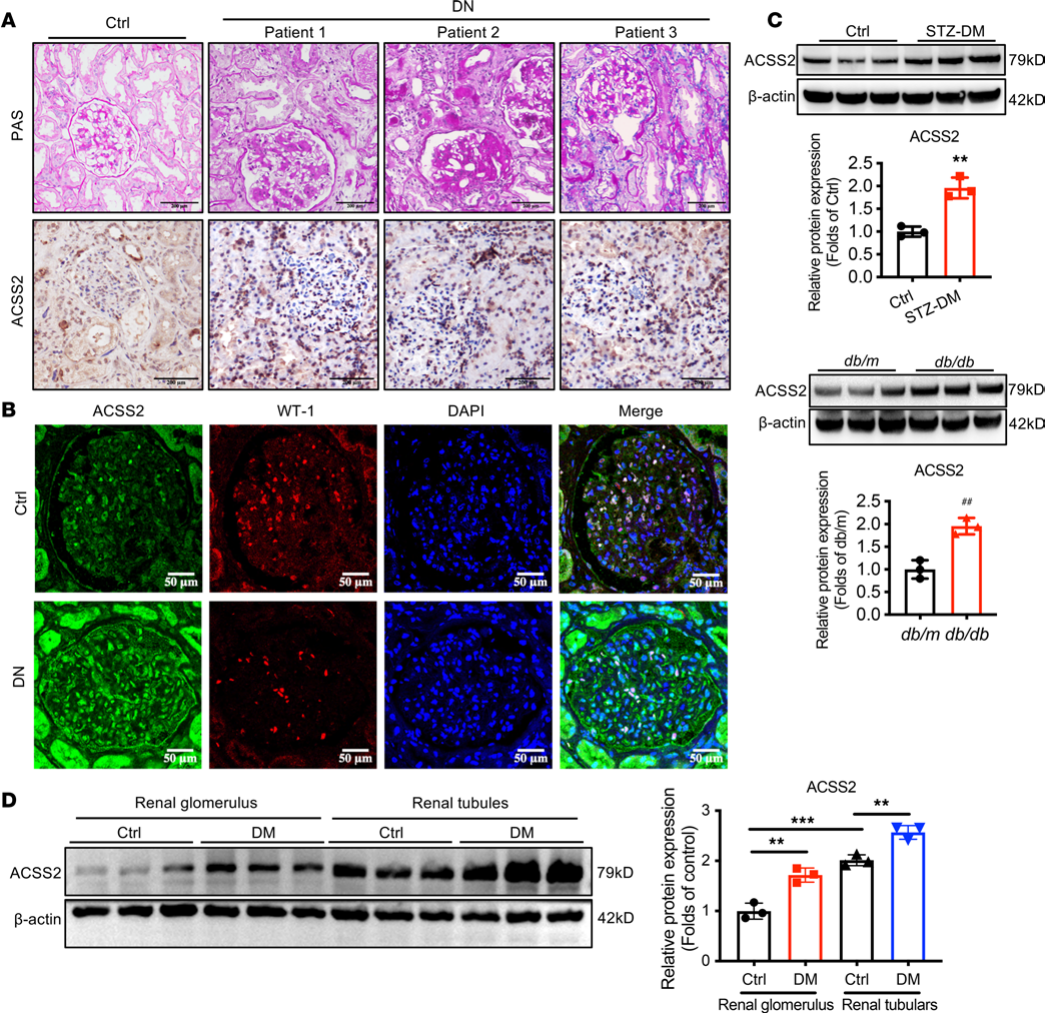
ACSS2 expression in kidneys of patients with DN and diabetic mice. (**A**) The periodic acid–Schiff (PAS) staining in human renal cortical tissues from normal kidney poles and individuals with histopathological lesions of DN. The representative images of immunohistochemical staining show the expression of ACSS2 in these individuals (original magnification ×400; scale bars, 50 and 100 μm). (**B**) The immunofluorescence staining showed the colocalization of podocyte-specific marker WT-1 (red) and ACSS2 (green) in the kidney of healthy individuals and DN participants. Nuclei were stained with DAPI (blue) (original magnification ×400; scale bars, 50 μm). (**C**) Relative ACSS2 protein expression levels in the kidneys from wild-type mice or STZ-induced diabetic mice for 12 weeks (*n* = 3 biological replicates, *t* test) or from *db/db* mice (*n* = 3 biological replicates, *t* test, mean ± SD, **P < 0.01 vs. Ctrl or ^##^*P* < 0.01 vs. *db/m* control). (**D**) Relative ACSS2 protein expression levels in the renal glomerulus and renal tubules from wild-type mice or 12-week STZ-induced diabetic mice (*n* = 3 biological replicates, *t* test, mean ± SD, **P < 0.01, ***P < 0.001. STZ, streptozotocin.

First, periodic acid–Schiff (PAS) staining showed the typical pathological change in kidney biopsies of patients with DN (Figure 1A). We then detected ACSS2 expression in the kidneys of patients with DN. Immunohistochemical staining showed an increased ACSS2 expression in diabetic kidneys. Furthermore, the expression of ACSS2 was increased in the podocytes of patients with DN, as shown by the colocalization of ACSS2 with Wilms’ tumor-1 (WT-1), a known podocyte-specific marker (Figure 1B). We further verified the increase of ACSS2 expression in the kidneys of *db/db* mice and the streptozotocin-induced diabetic mice as demonstrated by Western blotting (Figure 1C). Additionally, compared with the control group of mice, ACSS2 was indeed upregulated in both glomerulus and renal tubular tissues of diabetes mice (Figure 1D).

### Knockdown and pharmacological inhibition of ACSS2 attenuate high-glucose-induced podocyte injuries.

**Figure 2:**
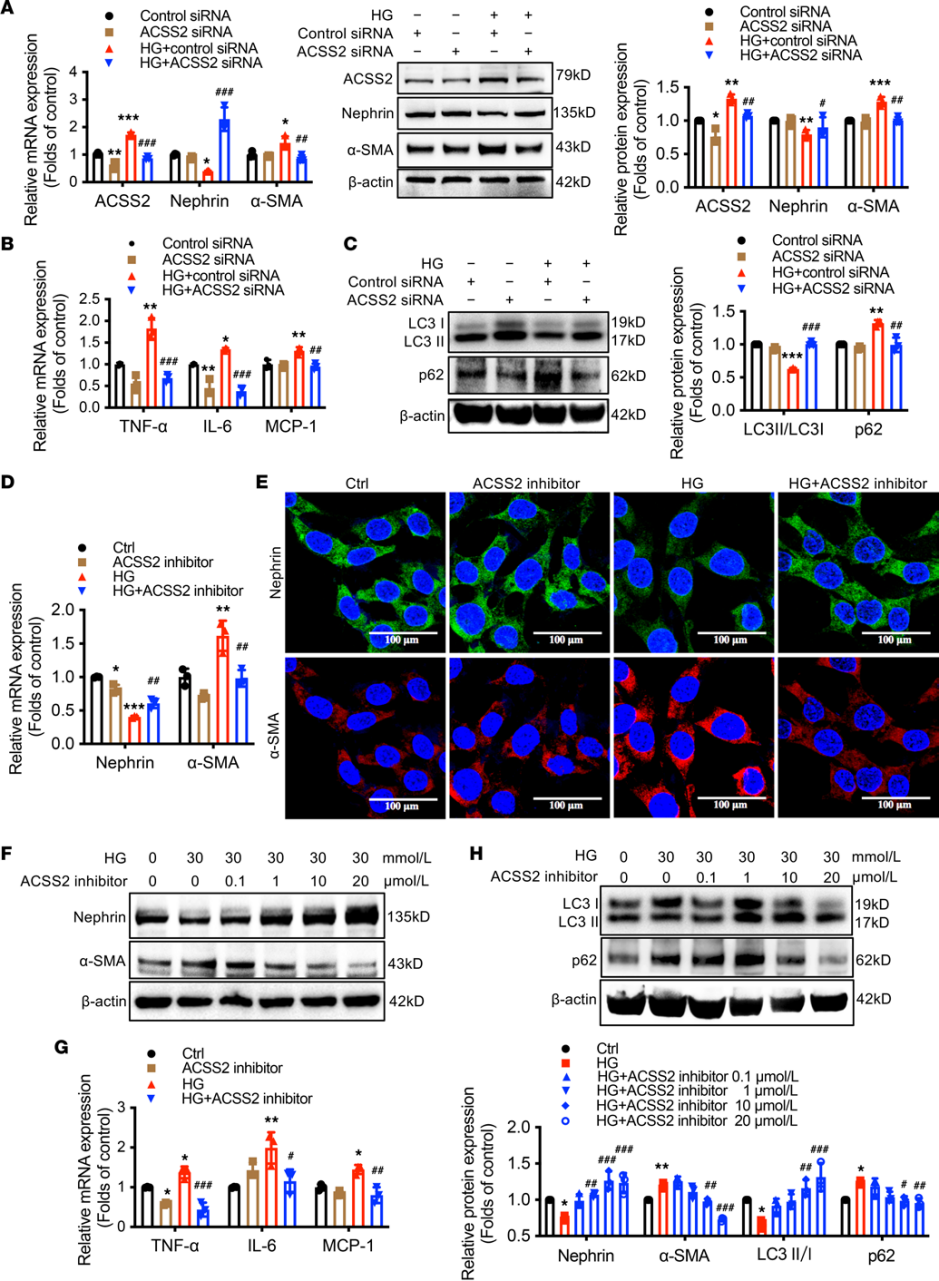
Knockdown or inhibition of ACSS2 attenuates HG-induced inflammation, prevents phenotypic transformation, and restores autophagy in podocytes. For **A**–**C**, HG-stimulated podocytes were treated with ACSS2 siRNA or control siRNA for 24 hours. Mean ± SD, *P < 0.05, **P < 0.01, ***P < 0.001 vs. control siRNA; ^#^*P* < 0.05, ^##^*P* < 0.01 ^###^*P* < 0.001 vs. HG + control siRNA (*n* = 3 biological replicates, 1-way ANOVA). (**A**) Real-time PCR and Western blotting analysis of ACSS2, nephrin, and α-SMA. (**B**) Real-time PCR analysis of mRNA expression of inflammatory factors (TNF-α, IL-6, and MCP-1). (**C**)Western blotting analysis of LC3 and p62. For **D**, **E**, and **G**, HG-stimulated podocytes with or without ACSS2 inhibitor (10 μmol/L) for 24 hours (mean ± SD, *P < 0.05, ***P < 0.001 vs. Ctrl; ^#^*P* < 0.05, ^##^*P* < 0.01, ^###^*P* < 0.001 vs. HG, *n* = 3). For **F** and **H**, HG-stimulated podocytes with or without ACSS2 inhibitor (10 μmol/L) for 24 hours (mean ± SD, *P < 0.05, **P < 0.01 vs. Ctrl; ^#^*P* < 0.05, ^##^*P* < 0.01, ^###^*P* < 0.001 vs. HG, *n* = 3). (**D**) Real-time PCR analysis of nephrin and α-SMA (*n* = 3 biological replicates, 1-way ANOVA). (**E**) Representative confocal microscopic images showing the expression of nephrin (green) and α-SMA (red). Nuclei were stained with DAPI (blue). Original magnification ×600; scale bars, 100 μm. (**F**) Western blotting analysis of nephrin and α-SMA (*n* = 3 biological replicates, 1-way ANOVA). (**G**) Real-time PCR analysis of the expression of inflammatory factors (TNF-α, IL-6, and MCP-1) (*n* = 3 biological replicates, 1-way ANOVA). (**H**) Western blotting analysis of LC3 and p62 (*n* = 3 biological replicates, 1-way ANOVA).

We next explored how ACSS2 activation mediated podocyte injury in DN. High-glucose (HG) stimulation induced ACSS2 mRNA expression (Figure 2A). Knockdown and pharmacological inhibition of ACSS2 ameliorated HG-induced decrease in nephrin expression and suppressed α–smooth muscle actin (α-SMA) expression in podocytes (Figure 2, A and D–F), indicating that ACSS2 activation promoted podocyte phenotypic transformation. Moreover, the knockdown and inhibition of ACSS2 decreased the mRNA expression of inflammatory cytokines TNF-α, IL-6, and monocyte chemoattractant protein-1 (MCP-1) in HG-treated podocytes (Figure 2, B and G). In addition, Figure 2, C and H, showed a downregulation of the LC3II/I ratio in HG-treated podocytes compared with the controls. Knockdown and inhibition of ACSS2 increased the LC3II/I ratio while decreasing the p62 level in podocytes. These results suggested that ACSS2 activation mediated HG-induced pleiotropic damage on podocytes.

### Protective effects of ACSS2 deletion on podocyte injury in STZ-induced diabetic mice.

**Figure 3:**
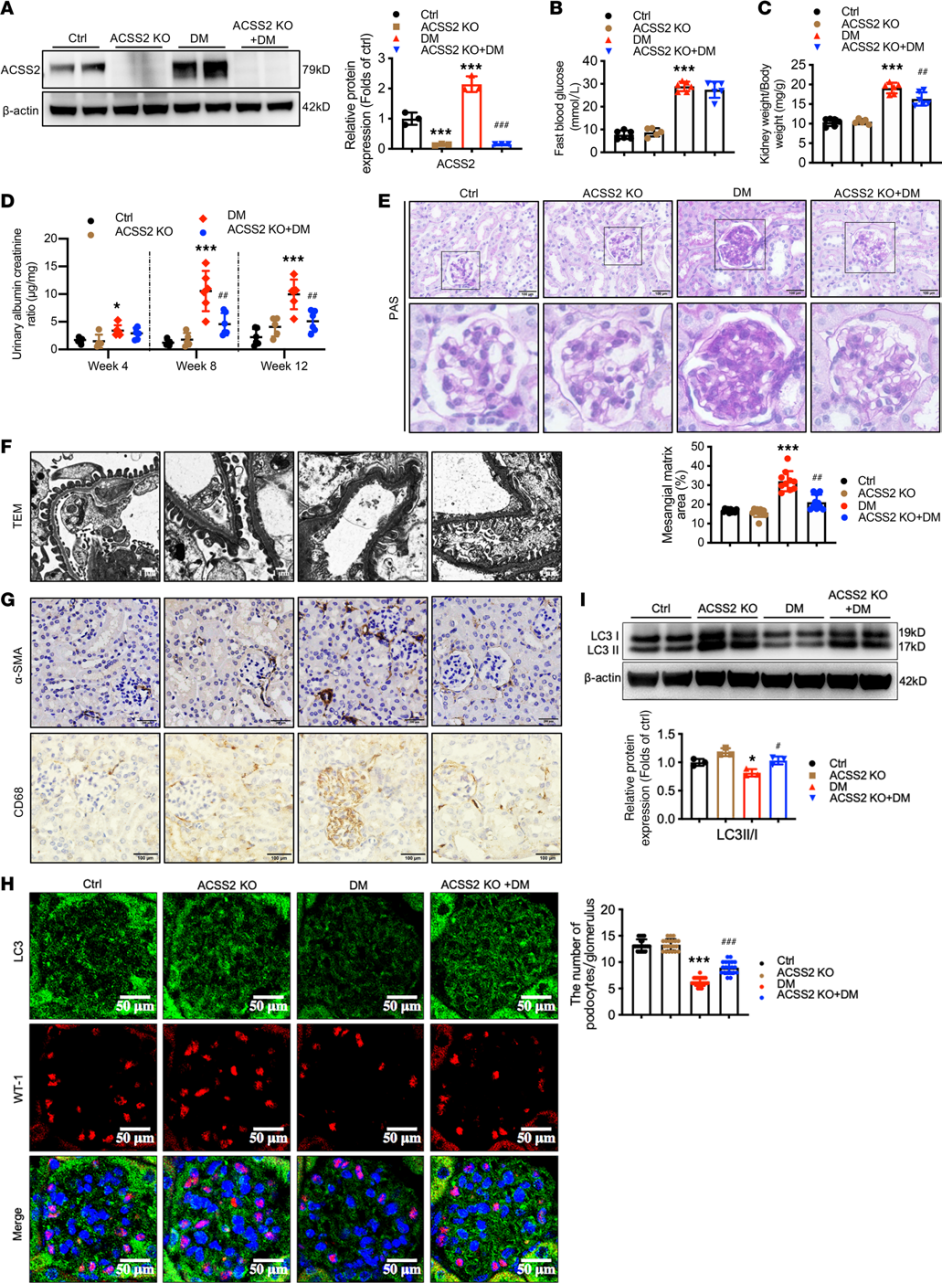
Protective effects of ACSS2 deletion on podocyte injury in STZ-induced diabetic mice. Four groups of mice of control (Ctrl) (*n* = 6), ACSS2 knockout (ACSS2 KO) (*n* = 5), diabetes (DM) (*n* = 6), and ACSS2 knockout with diabetes (ACSS2 KO + DM) (*n* = 6) were sacrificed at week 12. Data are expressed as mean ± SD or median with interquartile range, *P < 0.05, ***P < 0.001 vs. Ctrl; ^##^*P* < 0.01, ^###^*P* < 0.001 vs. DM). (**A**) Representative Western blotting images and densitometric analysis of ACSS2 protein expression in renal cortexes (*n* = 3 biological replicates, 1-way ANOVA). (**B**) The level of fasting blood glucose (*n* = 5–6 biological replicates, 1-way ANOVA). (**C**) The kidney weight–to–body weight ratio (*n* = 5–6 biological replicates, 1-way ANOVA). (**D**) Urinary albumin-to-creatinine ratio (ACR) was detected at weeks 4, 8, and 12 (*n* = 5–6 biological replicates, 1-way ANOVA). (**E**) Representative images of PAS-stained kidney sections (original magnification, ×400; scale bars, 100 μm). Bar graph analysis shows the quantification of the mesangial expansion area percentage (*n* = 10 biological replicates, 1-way ANOVA). (**F**) Representative images of glomerular ultrastructural change such as podocyte effacement and glomerular basement membrane (GBM) thickness observed by electron microscopy (original magnification ×40,000; scale bars, 1 μm). (**G**) Representative immunohistochemical staining images of α-SMA and CD68 (original magnification ×200, ×100; scale bars, 50 μm). (**H**) Representative confocal microscopic images showing the expression of LC3 (green) and WT-1 (red). Nuclei were stained with DAPI (blue). The quantifications of WT-1 (green) per glomerulus in the kidney (*n* = 20 biological replicates, 1-way ANOVA) were analyzed (original magnification ×400; scale bars, 50 μm). (**I**) Representative Western blotting images and densitometric analysis of LC3 protein expression (*n* = 3 biological replicates, 1-way ANOVA).

To further verify the role of ACSS2 in kidney injury in vivo, we generated global ACSS2-KO mice via CRISPR/Cas9 system. Quantitative real-time PCR analysis showed that ACSS2-KO mice displayed significantly reduced mRNA expression of ACSS2. The Western blotting analysis confirmed the gene ACSS2 KO efficiency in the kidneys (Figure 3A). At week 12 after STZ injection, blood glucose levels and kidney weight–to–body weight ratio were increased in STZ-induced diabetic mice compared with the controls. However, ACSS2 deletion ameliorated kidney hypertrophy in diabetic mice (Figure 3, B and C). Wild-type diabetic mice developed severe albuminuria at week 4 after STZ injection, which persisted up to week 12. By contrast, ACSS2-KO diabetic mice showed a much lower urinary albumin-to-creatinine ratio (ACR) at weeks 8 and 12 (Figure 3D). In addition, the histological changes in the diabetic mice kidney included mesangial matrix expansion (Figure 3E), glomerular basement membrane thickening, and podocyte foot process fusion (Figure 3F), which were mitigated in diabetic ACSS2-KO mice. There were also decreased α-SMA expression and CD68-positive macrophage infiltration in the kidneys of diabetic ACSS2-KO mice compared with wild-type diabetic mice (Figure 3G). Consistent with the results in vitro, ACSS2 deletion improved podocyte autophagy in diabetic mice. More LC3-positive puncta and increased LC3II/I ratio were present in podocytes of diabetic ACSS2-KO mice compared with wild-type diabetic mice, as demonstrated by immunofluorescence staining and Western blotting (Figure 3, H and I). These results further verified that ACSS2 activation suppressed autophagy in podocytes.

### ACSS2 activation inhibits autophagy through raptor-mediated mammalian target of rapamycin complex 1 pathway upregulation in podocytes.

**Figure 4:**
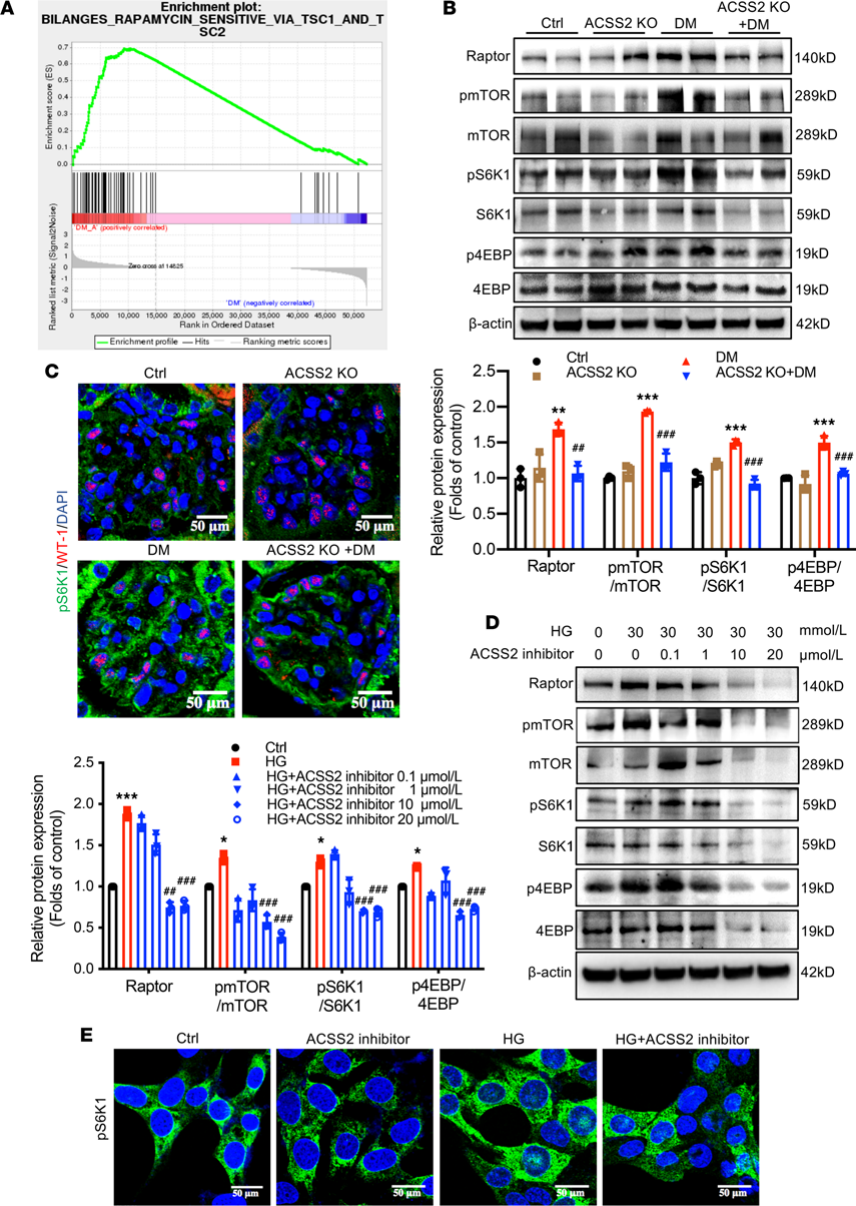
ACSS2 activation inhibits autophagy by upregulating the raptor-mediated mTORC1 pathway in podocytes. (**A**) Gene set enrichment analysis (GSEA) highlights strong rapamycin-sensitive pathway enrichment. (**B**) Western blotting analysis for the phosphorylation activation of raptor, mTOR, S6K1, and 4EBP in renal cortex tissues prepared from vehicle- or STZ-treated mice with or without ACSS2 KO after the 12-week observation period (mean ± SD, **P < 0.01, ***P < 0.001 vs. Ctrl; ^##^*P* < 0.01, ^###^*P* < 0.001, vs. DM, *n* = 3 biological replicates, 1-way ANOVA). (**C**) Representative confocal images illustrate p-S6K1 (green) and WT-1 (red) expression changes in kidney sections. Nuclei were stained with DAPI (blue) (original magnification ×400; scale bars, 50 μm, *n* = 3). (**D**) Western blotting analysis for raptor and the phosphorylation activation of mTOR, S6K1, and 4EBP in HG-stimulated podocytes treated with ACSS2-specific inhibitor (0, 0.1, 1, 10, 20 μmol/L) for 24 hours (mean ± SD, *P < 0.05, **P < 0.01, ***P < 0.001 vs. Ctrl; ^##^*P* < 0.01, ^###^*P* < 0.001 vs. HG, *n* = 3 biological replicates, 1-way ANOVA). (**E**) Representative confocal images illustrating p-S6K1 expression changes in HG-stimulated podocytes treated with ACSS2-specific inhibitor (10 μmol/L) for 24 hours. Nuclei were stained with DAPI (blue) (original magnification ×600; scale bars, 50 μm; *n* = 3).

Next, to investigate how ACSS2 deficiency ameliorated kidney injury in DN, we performed RNA-Seq analysis of the renal cortex from diabetic and diabetic ACSS2-KO mice. We identified 230 differentially expressed genes (DEGs) in the kidneys of diabetic ACSS2-KO mice, with 112 downregulated and 118 upregulated, compared with wild-type diabetic mice. Kyoto Encyclopedia of Genes and Genomes (KEGG) enrichment analysis demonstrated that compared with wild-type diabetic mice, diabetic ACSS2-KO mice were enriched for pathways associated with cellular metabolism. Moreover, gene set enrichment analysis (GSEA) showed that downregulated genes in diabetic ACSS2-KO mice were significantly enriched in rapamycin-sensitive signaling via tuberous sclerosis complex 1/2 (TSC1/2) (Figure 4A). Since the mammalian target of rapamycin complex 1 (mTORC1) pathway is downstream of TSC1/2 and negatively regulates autophagy in vivo and in vitro, levels of multiple mTORC1 signaling biomarkers were determined in STZ-induced diabetic mice and HG-treated podocytes. The levels of raptor, and phosphorylated (p-) mTOR, S6K1, and eukaryotic translation initiation factor 4E binding protein (4EBP), were significantly increased in renal cortices of STZ-induced mice, which were decreased in STZ-induced ACSS2-KO mice (Figure 4B). Immunofluorescence staining further validated a mild decrease in p-S6K1 levels in diabetic ACSS2-KO mouse glomerular podocytes (Figure 4C). In an in vitro study, HG treatment increased raptor expression and mTOR pathway phosphorylation in podocytes. However, phosphorylation of S6K1 and 4EBP was inhibited in podocytes by ACSS2 siRNA and ACSS2-specific inhibitor (Figure 4D). Immunofluorescence staining of p-S6K1 achieved similar results (Figure 4E). These findings suggested that HG-induced mTORC1 activation could be attributed to the upregulation of ACSS2, and the overactivation of ACSS2 in podocytes could be a key contributor to DN progression.

### The ACSS2 inhibitor ameliorates kidney injury in diabetic mice.

**Figure 5:**
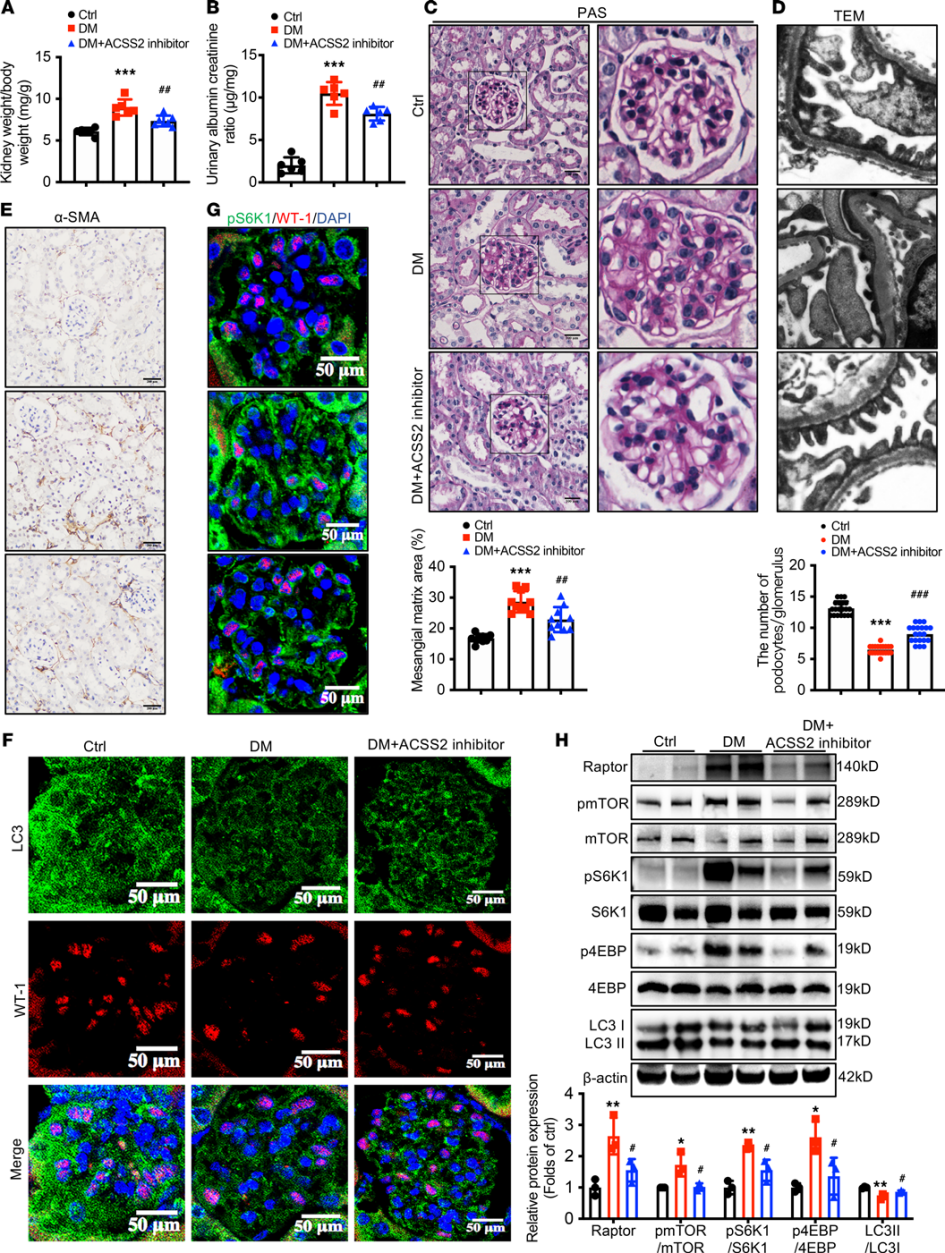
The ACSS2 inhibitor ameliorates kidney injury in diabetic mice. Diabetes was induced by intraperitoneal injection of STZ. The corresponding control mice were treated with vehicle (Ctrl). Diabetic mice were treated with vehicle (DM) or ACSS2 inhibitor (50 mg/kg) (DM+ACSS2 inhibitor). The mice were euthanized at week 12. Data are expressed as mean ± SD. *P < 0.05, **P < 0.01, ***P < 0.001 vs. Ctrl; ^#^*P* < 0.05, ^##^*P* < 0.01, ^###^*P* < 0.01 vs. DM, *n* = 6). (**A**) Kidney hypertrophy was determined by the ratio of kidney weight to body weight (*n* = 6 biological replicates, 1-way ANOVA). (**B**) Urinary ACR was measured by commercial ELISA for microalbuminuria and creatinine detection assay (*n* = 6 biological replicates, 1-way ANOVA). (**C**) Representative images of PAS-stained kidney sections (original magnification ×400; scale bars, 100 μm). Bar graph analysis shows the quantification of the mesangial expansion area percentage (*n* = 10 biological replicates, 1-way ANOVA). (**D**) Representative images of glomerular ultrastructural change such as podocyte effacement and GBM thickness were observed by electron microscopy (original magnification ×40,000; scale bars, 500 nm). (**E**) Representative immunohistochemical staining images of α-SMA in kidney sections (original magnification ×200; scale bars, 200 μm). (**F**) Representative confocal microscopic images showing the expression of LC3 (green) and WT-1 (red). Nuclei were stained with DAPI (blue). The quantifications of WT-1 (green) per glomerulus in the kidney (*n* = 20 biological replicates, 1-way ANOVA) were analyzed (original magnification ×400; scale bars, 50 μm). (**G**) Representative confocal microscopic images showing the expression of p-S6K1 (green) and WT-1 (red). Nuclei were stained with DAPI (blue) (original magnification ×400; scale bars, 50 μm). (**H**) Western blotting analysis for raptor and the phosphorylation activation of mTOR, S6K1, 4EBP, and LC3 in renal cortex tissues (*n* = 3 biological replicates, 1-way ANOVA).

To obtain additional evidence for the in vivo functional contribution of ACSS2 activation to DN pathogenesis, the mice were exposed to ACSS2 inhibitors by intragastric administration. ACSS2 inhibitor administration to diabetic mice exhibited no difference in body weight or blood glucose levels compared to diabetic mice. The kidney weight–to–body weight ratio was increased in diabetic mice, and the ACSS2 inhibition prevented these changes in diabetic mice (Figure 5A). In addition, compared with untreated diabetic mice, ACSS2 inhibitor administration significantly improved diabetic glomerular injury by reducing urinary ACR (Figure 5B), inhibiting glomerular mesangial expansion (Figure 5C), glomerular hypertrophy, and glomerular basement membrane thickening (Figure 5D). ACSS2 inhibition also reduced renal expression of α-SMA in diabetic mice (Figure 5E). We next determined if ACSS2 inhibition resulted in the downregulation of the mTORC1 pathway and the restoration of autophagy in podocytes. Immunofluorescence staining showed that renal WT-1 and LC3 coexpression levels in the diabetic kidneys were restored by ACSS2 inhibitor administration (Figure 5F). The p-S6K1 phosphorylation levels markedly decreased in the kidneys of ACSS2 inhibitor–treated diabetic mice compared with untreated diabetic mice (Figure 5G). Western blotting analysis showed the same tendency as in diabetic ACSS2-KO mice, and the ACSS2 inhibitor significantly downregulated the raptor/mTORC1 pathway in diabetic mice (Figure 5H). These data suggested that pharmacological inhibition ACSS2 could provide a protective role for the podocyte injury in DN through the improvement of podocyte autophagy.

### TFEB could be a downstream effector for the mTORC1 pathway activation in DN podocytes.

**Figure 6:**
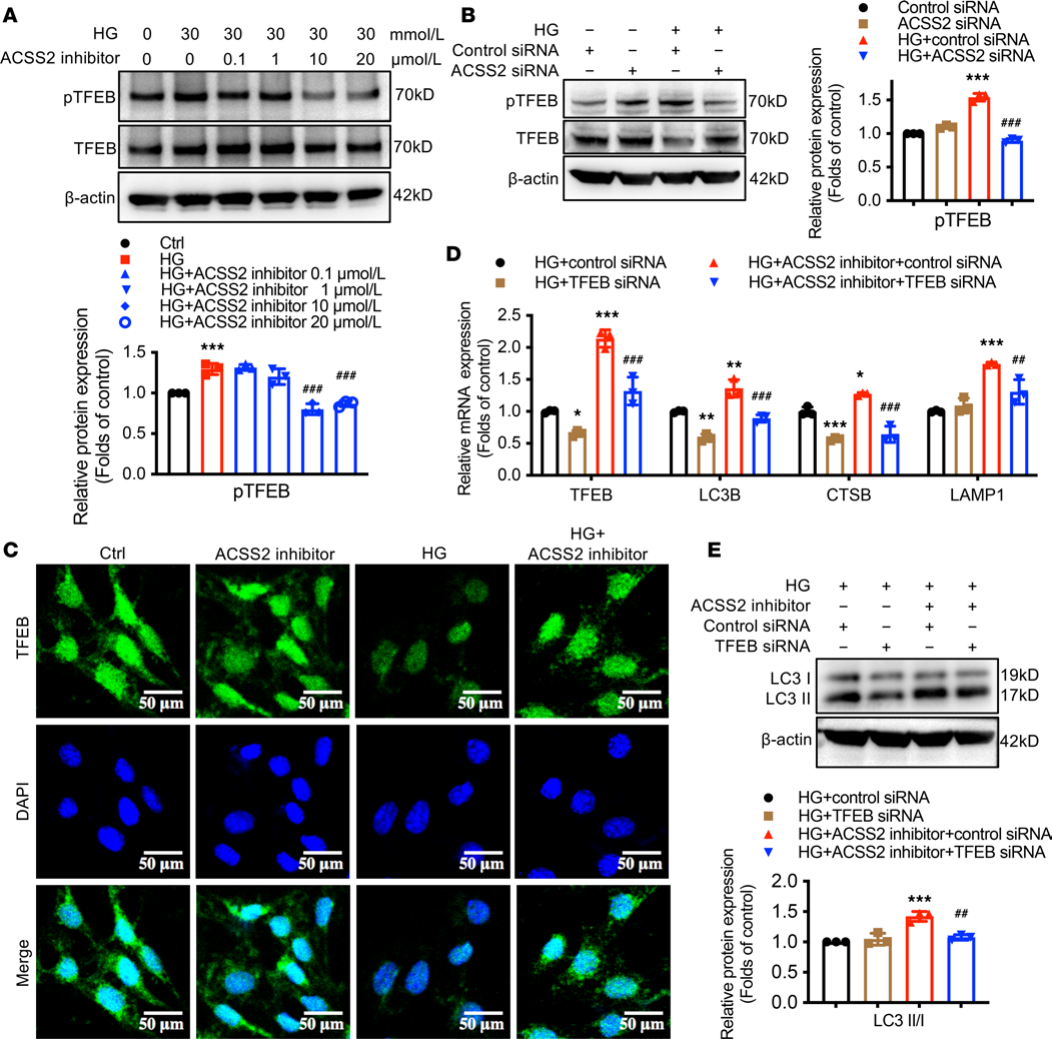
TFEB acts as a downstream effector for the activation of the mTORC1 pathway in HG-stimulated podocytes. (**A**) Western blotting analysis for p-TFEB and TFEB in HG-stimulated podocytes treated with ACSS2-specific inhibitor (0, 0.1, 1, 10, 20 μmol/L) for 24 hours (mean ± SD, ***P < 0.001 vs. Ctrl; ^###^*P* < 0.001 vs. HG, *n* = 3 biological replicates, 1-way ANOVA). (**B**) Western blotting analysis for p-TFEB and TFEB in HG-stimulated podocytes transfected with control siRNA or ACSS2 siRNA for 24 hours (mean ± SD, ***P < 0.001 vs. control siRNA; ^###^*P* < 0.001 vs. HG + control siRNA, *n* = 3 biological replicates, 1-way ANOVA). (**C**) Representative confocal microscopic images showing TFEB (green) expression and DAPI (blue) in HG-stimulated podocytes treated with ACSS2-specific inhibitor (10 μmol/L) for 24 hours (original magnification ×600; scale bars, 50 μm). (**D**) Real-time PCR analysis for the reverse effects of TFEB knockdown on the mRNA expression of TFEB, LC3B, CTSB, and LAMP1 in podocytes treated with ACSS2 inhibitor (mean ± SD, *P < 0.05, **P < 0.01, ***P < 0.001 vs. HG + control siRNA; ^##^*P* < 0.01, ^###^*P* < 0.01 vs. HG+ACSS2 inhibitor+control siRNA, *n* = 3 biological replicates, 1-way ANOVA). (**E**) Western blotting analysis for LC3 in HG-stimulated podocytes transfected with control siRNA or TFEB siRNA for 24 hours (mean ± SD, ***P < 0.001 vs. Ctrl; ^##^*P* < 0.01 vs. HG, *n* = 3 biological replicates, 1-way ANOVA).

Transcription factor EB (TFEB) is a master regulator for lysosomal biogenesis (20). Thus, we next investigated whether ACSS2-modulated autophagy inhibition could be due to impaired TFEB function in HG-treated podocytes. ACSS2 pharmacological inhibition and gene knockdown decreased TFEB phosphorylation in HG-treated podocytes (Figure 6, A and B). Immunofluorescence staining showed that TFEB localized in the cytosol and nuclei, and HG treatment significantly decreased the nuclear TFEB in podocytes. In contrast, the ACSS2 inhibitor restored nuclear TFEB levels (Figure 6C). We then evaluated the reverse effect of TFEB knockdown on podocyte autophagy inhibition under the HG condition. Results showed that TFEB siRNA treatment downregulated autophagy-related gene expression of LC3B, CTSB, and LAMP1 while decreasing the ratio of LC3II to LC3I in HG-mediated podocytes (Figure 6, D and E).

### ACSS2 activation contributes to raptor transcriptional activation via histone H3K9 acetylation in HG-treated podocytes.

**Figure 7:**
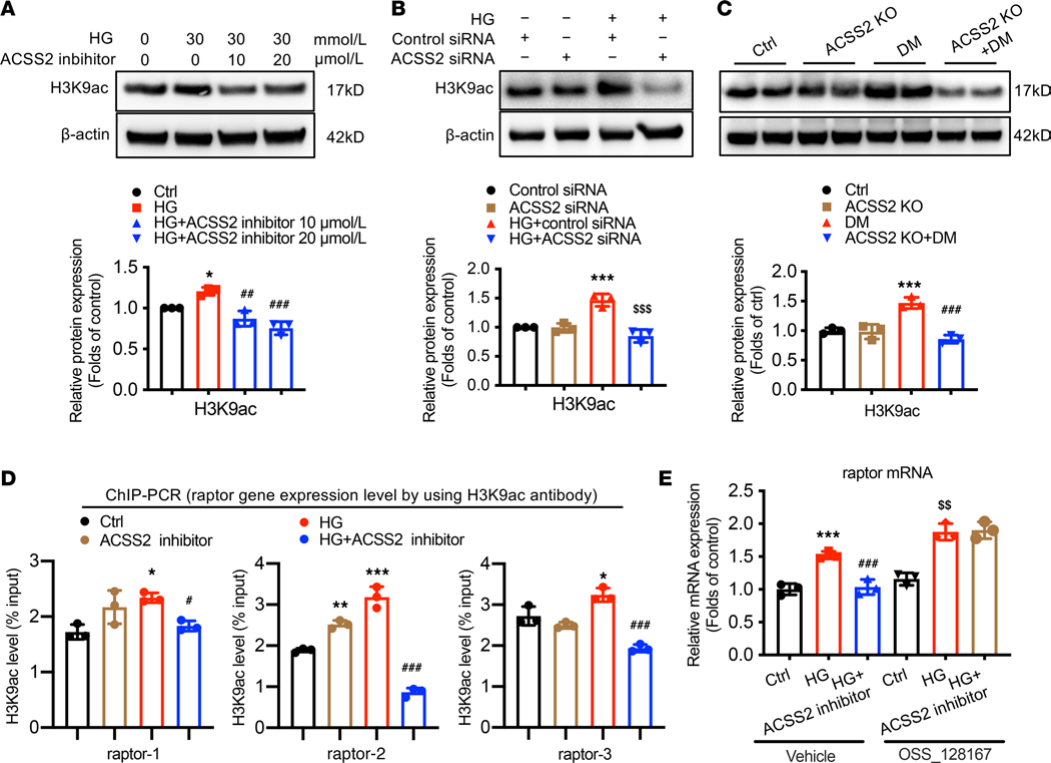
ACSS2 activation contributes to raptor transcriptional activation via H3K9ac in HG-treated podocytes. (**A** and **B**) Western blotting analysis showing the effects of inhibition (**A**) or gene knockdown (**B**) of ACSS2 on H3K9ac levels in podocytes treated with HG (mean ± SD, *P < 0.05 vs. Ctrl or ***P < 0.001 vs. control; ^##^*P* < 0.01, ^###^*P* < 0.001 vs. HG; ^$$$^*P* < 0.001 vs. control siRNA, *n* = 3 biological replicates, 1-way ANOVA). (**C**) Gene deletion of ACSS2 decreases the diabetes-induced upregulation of H3K9ac at raptor promoters in mouse kidneys (mean ± SD, ***P < 0.001 vs. Ctrl; ^###^*P* < 0.001 vs. DM, *n* = 3 biological replicates, 1-way ANOVA). (**D**) Chromatin immunoprecipitation (ChIP) analysis showing H3K9 acetylation levels in the promoters of raptor using antibodies to H3K9ac (mean ± SD, *P < 0.05, **P < 0.01, ***P < 0.001 vs. Ctrl; ^#^*P* < 0.05, ^###^*P* < 0.001 vs. HG, *n* = 3 biological replicates, 1-way ANOVA). (**E**) The reverse effects of OSS_128167 (a selective inhibitor that increases the acetylation of H3K9) to the transcriptional expression of raptor, which was inhibited by ACSS2 inhibitor on podocytes (mean ± SD, ***P < 0.001 vs. Ctrl; ^###^*P* < 0.001 vs. HG; ^$$^*P* < 0.01 vs. HG, *n* = 3 biological replicates, 1-way ANOVA).

To clarify potential mechanisms for the raptor/mTORC1 pathway upregulation in DN mediated by ACSS2, we evaluated the effect of HG treatment on histone acetylation in podocytes. Western blotting analysis showed that histone H3 acetylation at lysine 9 (H3K9ac) was increased in HG-treated podocytes, and ACSS2 inhibitor and ACSS2 siRNA attenuated H3K9ac level (Figure 7, A and B). In vivo studies achieved similar results (Figure 7C). Interestingly, ChIP analysis showed that ACSS2 activation increased the H3K9ac level in the raptor promoters in HG-treated podocytes (Figure 7D). These data suggested that ACSS2 activation could increase raptor mRNA expression by directly modifying H3K9 acetylation. We then examined possible mechanisms for the stimulatory effects of ACSS2-mediated epigenetic regulation of H3K9ac in raptor expression. Podocytes were pretreated with OSS_128167, a selective inhibitor that increases H3K9 acetylation, and then we treated podocytes with HG. Significant decreases in mRNA expression of raptor induced by ACSS2 inhibitor were reversed by OSS_128167 (Figure 7E).

## Discussion

Accumulating evidence has emphasized epigenetic modification in the regulation of kidney diseases. Previous studies have revealed the epigenetically mediated potential of ACSS2 in gene expression concerning energy metabolism (21, 22). While its role in renal diseases remains to be fully elucidated, in this study, for the first time, we demonstrated that ACSS2 was upregulated in the kidneys of individuals with DN and a diabetic mouse model. Moreover, ACSS2 was indeed upregulated in both glomerulus and renal tubular tissues of diabetic mice. Furthermore, pharmacological inhibition or gene knockout of ACSS2 reduced podocyte injury, decreased albuminuria, and mitigated glomerular pathological injury in DN. Mechanistically, ACSS2 mediated autophagy inhibition of podocytes through raptor/mTORC1 pathway modulation, thereby contributing to the DN progression. The most innovative finding of this study is that ACSS2 activation is involved in DN pathogenesis. ACSS2 gene KO could improve podocyte injury and proteinuria in diabetic mice, indicating that ACSS2 is pathogenic to DN. In addition, we also provided direct evidence that ACSS2 inhibitor administration in vivo had therapeutic potential for podocyte dysfunction and renal injury in diabetic mice.

Given the increased ACSS2 expression in the glomeruli of patients with DN, this study was designed to highlight and explore the role of ACSS2 in podocytes. mTOR is an evolutionarily conserved serine/threonine kinase that serves as the master regulator of anabolic metabolism and autophagy by responding to nutrients. The mTOR forms 2 functional complexes, termed mTORC1 and mTORC2. Activation of mTOR signaling is proposed as a cause of diabetes-associated glomerular injury as mTOR regulates the podocyte size (23, 24). Previous studies have emphasized that the typical characteristics of diabetes are mTORC1 overactivation and autophagy dysfunction (25, 26). Thus, an approach to preventing mTORC1 activation may ameliorate podocyte injury and delay the onset and development of glomerular disease (27). Interestingly, ACSS2/mTOR plays a vital role in controlling the nucleocytoplasmic acetyl-CoA pool (28). In mouse embryonic fibroblasts, ATP-citrate lyase deletion resulted in ACSS2 upregulation and a compensatory increase of acetate production, which partially rescued lipid droplet synthesis and peroxisome proliferator–activated receptor γ2 expression (28). Thus, this study is the first to our knowledge to establish a connection between ACSS2 and mTORC1. Furthermore, we also found a previously neglected tendency in ACSS2-dependent mTORC1 signaling toward autophagy inhibition that provides a plausible mechanistic basis for why ACSS2 activation impairs podocyte autophagy.

High basal autophagy activity and an intact autophagy-lysosome system are crucial in maintaining podocyte homeostasis (29). In contrast, hyperglycemia-suppressed podocyte autophagy and autophagy dysregulation further contribute to DN progression (30, 31). Studies have implicated that autophagy defects are involved in the pathogenesis of glomerular diseases (32–34). Podocyte-specific autophagy deficiency and aberrant accumulation of the autophagy substrate p62 deteriorated podocyte loss and proteinuria in diabetic mice (35), indicating that impaired podocyte autophagy is involved in DN. Podocyte-specific autophagy-related protein 5 deletion contributes to endoplasmic reticulum stress, histologic glomerulosclerosis, and proteinuria in aging mice (36). In addition, diabetic patients with overt proteinuria exhibited insufficient podocyte autophagy and podocyte loss. These findings suggested that diabetic patients with proteinuria may have pathogenic factors leading to podocyte dysfunction and autophagy disorder. Otherwise, during diabetic metabolic stress, enhancing autophagy activity may benefit renal injury. A highlighted observation of this study is the ACSS2 role in podocyte autophagy. We found that podocyte autophagy in STZ-induced diabetic mice was markedly reduced, while autophagy in podocytes was enhanced after the treatment with the ACSS2 inhibitor. Our findings are consistent with previous studies showing that autophagy homeostasis maintenance in podocytes plays an important role in preventing DN progression.

The most widely understood ACSS2 function is its control of acetyl-CoA production from acetate (12). Previous studies have shown that glucose deprivation leads to ACSS2 phosphorylation and nuclear localization. ACSS2 in the nucleus induces lysosomal and autophagic gene expression by regulating histone H3 acetylation (11) and promotes lysosomal biogenesis, autophagy, and brain tumorigenesis (13). These results emphasized the importance of ACSS2-mediated histone acetylation in cell energy homeostasis. In addition, the glucose/pyruvate/acetate metabolic axis is coupled with the mitochondrial metabolism of mammalian cells in limited energy environments (37). During this process, ACSS2 is necessary for the enzymatic catalysis of endogenous acetate into acetyl-CoA. This study revealed the direct relationship between glucose metabolism, ACSS2 activation, and podocyte autophagy hemostasis. However, the action and mechanism of ACSS2 during overnutrition (such as in an HG environment) have been enigmatic.

Collectively, our findings elucidate a lysosome-metabolic link that limits podocyte autophagic degradation, suggesting that catalytic action via ACSS2 is a major source of cytosolic acetyl-CoA that maintains podocyte metabolic homeostasis. The deletion and inhibition of ACSS2 prevent the progression of DN by maintaining podocyte function through epigenetic regulation of raptor/mTORC1 signaling. Improving autophagy homeostasis by inhibiting ACSS2-mediated acetyl-CoA production might lead to an innovative therapeutic target for DN.

## Methods

### Human kidney biopsy specimens.

Renal biopsies were collected from the routine clinical diagnostic investigation at the Department of Nephrology, Nanjing Drum Tower Hospital, the Affiliated Hospital of Nanjing University Medical School. DN was diagnosed based on histopathological examination. Paraffin sections of renal biopsies were examined in 10 patients with DN. Control samples were obtained from para-carcinoma kidney tissues from 5 patients who underwent renal carcinoma resection, with no clinically obvious evidence of renal injury manifestations.

### Generation of ACSS2-KO mice.

C57BL/6J background heterozygous ACSS2-KO mice (Strain T017245) were obtained from GemPharmatech Co., Ltd. Transgenic mice were genotyped by quantitative real-time PCR. All experiments were performed with genetically appropriate littermate controls.

### STZ-induced mouse model.

Experimental diabetes was induced by STZ (MilliporeSigma, S0130) intraperitoneal injection in 8-week-old, wild-type, male C57BL/6J and ACSS2-KO mice. STZ dissolved in sodium citrate buffer (pH 4.5) was injected for 5 consecutive days in a dose of 50 mg/kg body weight. Control mice received vehicle citrate buffer. Two weeks after STZ treatment, mice with a fasting glucose concentration greater than 16.7 mmol/L were considered diabetic. STZ-treated mice that developed diabetes mellitus were enrolled in the study. The mice were divided into 4 groups: control (Ctrl) (*n* = 6), ACSS2 knockout (ACSS2-KO) (*n* = 5), diabetes (DM) (*n* = 6), and ACSS2 knockout with diabetes (ACSS2-KO + DM) (*n* = 6). The mice were humanely sacrificed at 12 weeks after STZ treatment.

### Intervention of ACSS2 inhibitor for diabetic mouse model.

After successfully inducing the diabetic mouse model, we administered the ACSS2 inhibitor (Selleck Chemicals, S8588) by gavage at a dose of 50 mg/kg (of mouse body weight) once a day for a follow-up course of 12 weeks. Control mice received a vehicle solution. The mice were divided into 3 groups: Ctrl (*n* = 6), DM (*n* = 6), and diabetes with ACSS2 inhibitor (DM + ACSS2 inhibitor) (*n* = 6). Body weight and fasting blood glucose concentration were monitored during the experimental period. The mice were humanely sacrificed at 12 weeks after STZ treatment.

### ELISA detection of microalbuminuria.

For diabetic mice, urine samples were collected from all mice in weeks 4, 8, and 12. Microalbumin and urinary creatinine levels were measured with Albumin Mouse ELISA Kit (Elabscience, E-EL-M0792c) and Creatinine Assay Kit (Jiancheng, C011-2-1) according to the manufacturer’s instructions. Results are expressed as the urine microalbumin-to-creatinine ratio.

### Mouse histopathology assessment.

The 4 μm mouse kidney sections were paraformaldehyde-fixed and paraffin-embedded for PAS and ACSS2 immunohistochemical staining. The PAS staining was quantified as positive area per glomerulus area using ImageJ software (NIH), which was also used to evaluate the percentage of glomerular mesangial expansion as previously described (38).

### Transmission electron microscopy.

Electron microscopic sample handling and detection were performed by the Electron Microscopic Central Laboratory of Southeast University Medical school as previously described (39). Kidney tissue samples were fixed in 2.5% glutaraldehyde in 0.1 mol/L phosphate buffer and processed for routine electron microscopy.

### Cell culture, siRNA transfection, and inhibitors.

Immortalized mouse podocytes were a gift from Ai Hua Zhang from the Children’s Hospital of Nanjing Medical University (Nanjing, China). Mouse podocytes were cultured in RPMI 1640 medium (Wisent) with 10% fetal bovine serum and 10 U/mL recombinant mouse γ-interferon (γ-IFN; PeproTech) at 33°C for proliferation. Podocytes were then transferred to nonpermissive conditions at 37°C without γ-IFN for at least 7 days for differentiation (40). Podocytes were incubated in RPMI 1640 with 10% FBS in the presence of 30 mmol/L d-glucose (MilliporeSigma) as the final concentration. The cells were exposed to 11 mmol/L d-glucose as the control. According to the procedure recommendation, ACSS2 siRNA transfection was performed with Lipofectamine 2000 from Thermo Fisher Scientific. ACSS2 inhibitor (Selleck Chemicals, S8588) or DMSO as control was added to podocytes before HG treatment. The sequences of control siRNA and ACSS2 siRNA are listed in Supplemental Table 3; https://doi.org/10.1172/jci.insight.165817DS1

### RNA extraction and quantitative real-time PCR analysis.

Total RNA was extracted from renal cortical tissue or podocytes with Trizol (Vazyme, R401-01) and reverse-transcribed into cDNA. Quantitative real-time PCR was performed using SYBR Green reagent (Vazyme, Q711-02) on the ABI 7300 PCR System (Applied Biosystems). The primers for mRNA detection are summarized in Supplemental Table 1. The relative expression levels of target genes were calculated as ΔΔCt.

### Western blotting.

Kidney tissues and cell culture samples were homogenized in RIPA lysis buffer. The protein contents were measured by using a bicinchoninic acid assay. A total of 30~50 μg of protein was resolved by SDS-PAGE, transferred to PVDF membranes, and probed with specific antibodies. β-Actin was used as an internal control. The following primary antibodies were used: anti-ACSS2 (ab133664), anti-p62 (ab56416), anti-S6K1 (ab32529), and anti–p-S6K1 (ab59208) purchased from Abcam; anti-nephrin (sc-376522) purchased from Santa Cruz Biotechnology; anti–α-SMA (14395-1-AP) and anti-LC3 (14600-1-AP) purchased from Proteintech; and anti-Raptor (no. 2280), anti-mTOR (no. 2983), anti–p-mTOR (no. 5536), anti-4EBP (no. 9644), anti–p-4EBP (no. 2855), and anti-H3K9ac (no. 9649) purchased from Cell Signaling Technology. The densitometric analysis was performed using ImageJ.

### Immunofluorescence staining and confocal microscopy.

Podocytes were washed with PBS, fixed with 4% paraformaldehyde, and permeabilized with 0.1% Triton X-100. Podocytes were blocked with 5% normal goat serum, then incubated with primary antibodies at 4°C overnight, followed by incubation with Alexa Fluor 488– or 594–conjugated secondary antibodies at 37°C for 60 minutes. Coverslips were sealed before imaging. Images were obtained using a confocal microscope (FV3000, Olympus).

### ChIP assay.

ChIP was performed with the Simple ChIP Enzymatic Chromatin IP Kit following the manufacturer’s instructions (41). Briefly, podocytes were fixed with 1% formaldehyde and we quenched formaldehyde by glycine. After cell lysis, chromatin was subsequently fragmented by enzymatic digestion and sonication, then subjected to IP with an antibody against H3K9ac at 4°C overnight and incubated with protein G magnetic beads at 4°C for 2 hours. The immunoprecipitate was washed and subjected to elution. Eluted DNA and input DNA were reversed by cross-linking, digested with proteinase K, and then purified with spin columns. The precipitated DNA fragments’ relative abundance was analyzed by real-time PCR. Primer pairs used for raptor ChIP-PCR are listed in Supplemental Table 2. All ChIP signals were calculated by the 2^-ΔΔCt^ method and normalized to the corresponding input signals: ChIP signals (% input) = 2^(Ct^ ^of^ ^input^^–^^Ct^ ^of^ ^IP^ ^sample)^ × 100.

### RNA-Seq.

The RNA-Seq transcriptome library of renal tissues was conducted according to TruSeq RNA sample preparation methods and then sequenced with the Illumina NovaSeq 6000 sequencer. The DEGs were identified based on a FDR-adjusted *P* < 0.05 and fold-change > 2 or < 0.5. Additionally, KEGG functional enrichment analysis and GSEA were performed to identify DEGs significantly enriched in Gene Ontology terms and metabolic pathways at a Bonferroni-corrected *P* < 0.05.

### Statistics.

Data are presented as mean ± SD or median with an interquartile range. To analyze the difference between the 2 groups, we used the standard 2-tailed *t* test when the assumptions (equal variance and normal distribution) were met. Otherwise, the nonparametric Mann-Whitney *U* test was used. For results with more than 2 groups, 1-way ANOVA followed by the Bonferroni post hoc test was used to analyze the difference among the groups. The results were analyzed by GraphPad Prism 7.0 software. *P* < 0.05 was considered statistically significant.

### Study approval.

The human ethics committee of Southeast University approved all human studies, and the participants provided informed consent. All animal experimental procedure protocols were approved by the institutional research ethics committee of the Nanjing Drum Tower Hospital, the Affiliated Hospital of Nanjing University Medical School, and procedures were performed in accordance with the NIH guidelines (*Guide for the Care and Use of Laboratory Animals*) (National Academies Press, 2011). This study complied with all relevant ethical regulations for animal testing and research, and the Southeast University Medical School Committee on Animal Care approved all animal studies.

### Data availability.

For the data generated in this manuscript, the RNA-sequencing data have been submitted to National Center for Biotechnology Information BioProject database with the identifier PRJNA982547 (https://www.ncbi.nlm.nih.gov/bioproject/PRJNA982547). All other data generated or analyzed during this study are included in this manuscript, the Supporting Data Values file, and its supplemental files. The data sets will be available from the corresponding author upon reasonable request.

## Author contributions

KLM and JL designed the study. JL, PPC, JXZ, and XQL performed the experiments and established the gene-modified mouse models. The data were analyzed by LL, GHW, XQL, TTJ, MYW, and WTL. KLM and XZR drafted and revised the manuscript. KLM and JL have verified the underlying data. All authors had access to the data, reviewed the manuscript, and approved the final version for submission.

## Supplementary Material

Supplemental data

Supporting data values

## Version 1 10/23/2023

Electronic publication
