# Supplementary material for: Activation of acetyl-CoA synthetase 2 mediates kidney injury in diabetic nephropathy
Source: JCI Insight. 2023 Oct 23;8(20):e165817. doi: 10.1172/jci.insight.165817 (PMC10619493; doi:10.1172/jci.insight.165817)
Supplement: Supplemental data [file jciinsight-8-165817-s161.pdf]

Table 1. the primer sequences of genes

Gene	Forward primer (5' to 3')	Reverse primer (5' to 3')
ACSS2	TGCTTCTTTCCCATTCTTCG	GCCCGTGATCCAGTAATAGC
nephrin	GCCTCCTGACCATTGCTAA	TTTCCACTCCAGTCCTACCG
α-SMA	CAGCAAACAGGAATACGACGAA	AACCACGAGTAACAAATCAAAGC
TNF-α	CTCCTCCACTTGGTGGTTTGT	GGTGCCTATGTCTCAGCCTCT
MCP-1	TGCCCTAAGGTCTTCAGCAC	AAGGCATCACAGTCCGAGTC
IL-6	CCTTCCAAAGATGGCTGAAA	AGCTCTGGCTTGTTCCTCAC
TFEB	ACAGTCTCCGTTCCATCACC	GGAGCAGGGAGTCATCTAGG
LAMP1	TGCACACAGGATGGACCTT	TGTGTCATTTGGGCTGATGT
CTSB	TACCTCGCATAGCCAGTGAA	ACTCCCAGAGATCGGCTTG
LC3B	CCTTCCAAAGATGGCTGAAA	AGCTCTGGCTTGTTCCTCAC
raptor	GGCAAGGGCTCTCAGACAT	GGTGCGAATCACAAGACAGA

Table 2. the primer sequences of raptor for ChIP analysis

Gene	Primer pairs (5' to 3')		Primer fragment size(bp)
raptor-1	Forward	TTGGGACAAGGTGGAAGCA	146
	Reverse	CATTATCCTCCTAGTCTCAGAGCAGT	
raptor-2	Forward	TAGACCAGGCTGACCTCCAAC	248
	Reverse	GCATTTTCTCAGTCTTAGGTTCCT	
raptor-3	Forward	ACTAACAGAGGCTCCCCTAACAC	276
	Reverse	AACCAGGGTGCGAGTGAGA	

Table 3. RNA sequences for siRNA interference

siRNA	sense (5' to 3')	antisense (5' to 3')
ACSS2<br>siRNA	GCCCAUUCCUCCAGUAC<br>AATT	UUGUACUGGAGGAAUGGGC<br>TT
Control<br>siRNA	UUCUCCGAACGUGUCAC<br>GUTT	ACGUGACACGUUCGGAGAA<br>TT
